# No Evidence for XMRV in German CFS and MS Patients with Fatigue Despite the Ability of the Virus to Infect Human Blood Cells *In Vitro*

**DOI:** 10.1371/journal.pone.0015632

**Published:** 2010-12-22

**Authors:** Oliver Hohn, Kristin Strohschein, Alexander U. Brandt, Sandra Seeher, Sandra Klein, Reinhard Kurth, Friedemann Paul, Christian Meisel, Carmen Scheibenbogen, Norbert Bannert

**Affiliations:** 1 Centre for Biological Security 4, Robert Koch-Institute, Berlin, Germany; 2 Institute for Medical Immunology, Charité - Universitätsmedizin Berlin, Berlin, Germany; 3 NeuroCure Clinical Research Center (NCRC), Charité Universitätsmedizin Berlin, Berlin, Germany; 4 Robert Koch-Institute, Berlin, Germany; 5 Centre for Retrovirology, Robert Koch-Institute, Berlin, Germany; University of Cambridge, United Kingdom

## Abstract

**Background:**

Xenotropic murine leukemia virus-related virus (XMRV), a novel human retrovirus originally identified in prostate cancer tissues, has recently been associated with chronic fatigue syndrome (CFS), a disabling disease of unknown etiology affecting millions of people worldwide. However, several subsequent studies failed to detect the virus in patients suffering from these illnesses or in healthy subjects. Here we report the results of efforts to detect antibody responses and viral sequences in samples from a cohort of German CFS and relapsing remitting multiple sclerosis (MS) patients with fatigue symptoms.

**Methodology:**

Blood samples were taken from a cohort of 39 patients fulfilling the Fukuda/CDC criteria (CFS), from 112 patients with an established MS diagnosis and from 40 healthy donors. Fatigue severity in MS patients was assessed using the Fatigue Severity Scale (FSS). Validated Gag- and Env-ELISA assays were used to screen sera for XMRV antibodies. PHA-activated PBMC were cultured for seven days in the presence of IL-2 and DNA isolated from these cultures as well as from co-cultures of PBMC and highly permissive LNCaP cells was analyzed by nested PCR for the presence of the XMRV *gag* gene. In addition, PBMC cultures were exposed to 22Rv1-derived XMRV to assess infectivity and virus production.

**Conclusion:**

None of the screened sera from CFS and MS patients or healthy blood donors tested positive for XMRV specific antibodies and all PBMC (and PBMC plus LNCaP) cultures remained negative for XMRV sequences by nested PCR. These results argue against an association between XMRV infection and CFS and MS in Germany. However, we could confirm that PBMC cultures from healthy donors and from CFS patients can be experimentally infected by XMRV, resulting in the release of low levels of transmittable virus.

## Introduction

Retroviruses are able to induce immunodeficiency, malignant transformation and neurologic diseases. In addition to HTLV and HIV, evidence for a third exogenous human retrovirus was published in 2006. This previously unknown gammaretrovirus was identified in prostate cancer patients and named xenotropic murine leukemia virus-related virus (XMRV) based on its high sequence similarity to endogenous xenotropic murine leukemia viruses [1]. In the initial report and in a recently published study [2] XMRV infection strongly correlated with a presumably impaired antiretroviral response due to a genetic polymorphism in the RNASEL gene encoding a type I interferon-induced endonuclease. A potential role of XMRV in the etiology of prostate cancer was further strengthened by a report from Schlaberg and coworkers identifying the virus in the epithelial tumor cells of 27% of sporadic prostate cancer patients [3] although no association with the RNASEL polymorphism was found but rather a positive correlation with tumor grade. In contrast to these reports involving American patients, studies using European cohorts found no [4], [5] or a very low prevalence [6] of XMRV in prostate cancer tissues.

In a recent *Science* publication, XMRV was linked to a completely different disease: chronic fatigue syndrome (CFS), also known as myalgic encephalomyelitis (ME) [7]. The retrovirus was detected in 68 of 101 patients tested and in 3.7% of healthy controls. Infection of blood cells was demonstrated, virus was transmittable to indicator cells or to fresh PBMC and plasma samples from CFS patients were shown to contain virus-specific antibodies [7]. CFS is a disease characterized by a long-lasting disabling fatigue accompanied by physical symptoms that resemble a severe flu-like illness [8]. Several viral (including retroviral) or microbial agents have been suggested to be involved in CFS, particularly as the onset of symptoms in many patients begin with an infectious illness. Furthermore, CFS can occur at any age, affecting both children and adults but for unknown reasons has a higher prevalence in women than in men [9].

Multiple Sclerosis (MS) is a common chronic neuroimmunologic disorder, whose etiology is not yet fully understood. Characterized by lymphocytic infiltration and damage of myelin sheaths and axons in the early stages, the disease can progress to extensive neurodegeneration with severe disability in later stages [10]. Although fatigue is one of the most common symptoms in MS, being reported by more than 90% of patients [11], the biological cause of MS related fatigue remains unknown. It shares many of the hallmark symptoms of CFS and has a substantially negative impact on the quality of life of those affected [12].

Due to the potential implication of these findings, not only for the estimated 17 million people worldwide suffering from CFS but also for public health, including blood safety, a thorough investigation of the overall prevalence and association of XMRV with CFS is crucial. Following shortly the initial publication by Lombardi et al. [7], three European studies failing to detect XMRV in blood samples from independent cohorts of CSF patients were published [13], [14], [15]. However, these reports are severely criticized because the PCR analyses were performed using DNA from unstimulated PBMCs: Gammaretroviruses such as XMRV are assumed to productively infect only dividing cells [16] and activation of the cells allowing virus proliferation *in vitro* might therefore be necessary for successful detection.

In the present report we have analyzed samples from well-characterized German cohorts of CFS patients and MS patients with fatigue for evidence of XMRV. In addition, the ability of XMRV to infect and establish productive replication in PBMC from CFS patients and healthy blood donors was evaluated.

## Results

### XMRV antigen ELISA

**Figure 1:**
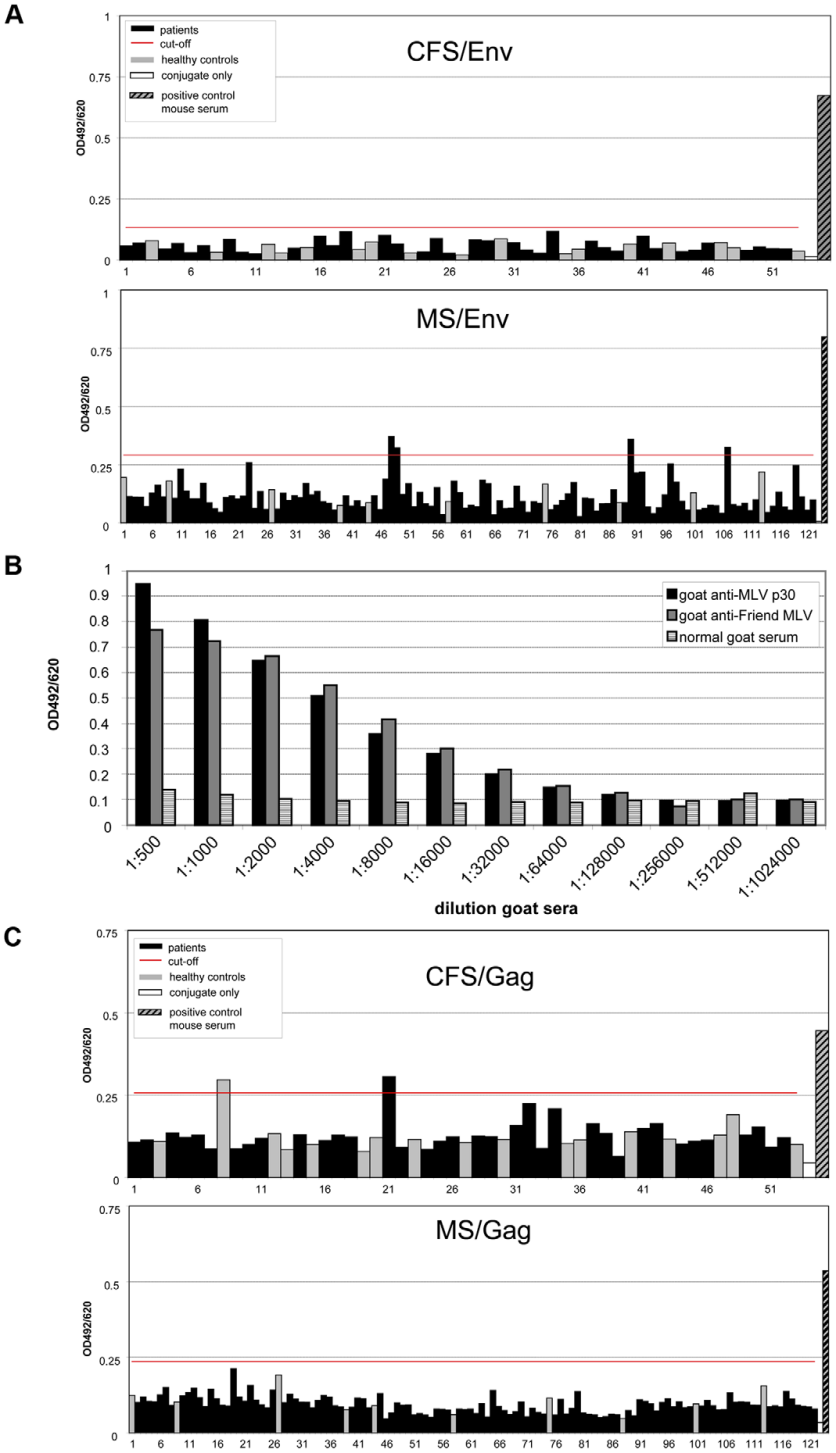
Serological assays. ELISAs with recombinant XMRV proteins were used to detect specific humoral responses. The cut-off was calculated as the mean of all sera from healthy controls plus three times the standard deviation. (A) Results of an XMRV Env antibody ELISA with sera from 36 CFS patients and 17 healthy controls (upper panel) and 112 MS patients and 10 healthy controls (lower panel) identified after unblinding. Sera were incubated at a dilution of 1∶200. (B) Titration of positive control goat sera versus recombinant Gag protein captured by the monoclonal anti-MLV Gag antibody R187. (C) Capture ELISA for the detection of XMRV anti-Gag antibodies in CFS (upper panel) and MS (lower panel) patient sera and healthy controls (gray bars in both panels). Human sera were diluted 1∶200 in blocking buffer.

Infection with a novel retrovirus such as XMRV would be expected to result in the induction of an immune response, particularly with antibodies specific for the viral Env and Gag proteins. Envelope specific antibodies have recently been shown to be present in the plasma of XMRV-infected CFS patients and sera from XMRV-positive prostate cancer patients were shown to neutralize the virus [2], [7]. We therefore analyzed sera from 36 CFS patients, 112 multiple sclerosis patients and 27 healthy individuals for serological evidence of XMRV infection (Table 1). Initially, an ELISA using recombinant XMRV Env proteins as antigen was used as described previously [5]. Although panels of XMRV positive human specimens were not available this assay has recently been validated in a blinded fashion using goat polyclonal anti-MuLV gp70 antisera and corresponding goat control sera [17]. At a 1∶200 dilution, none of the CFS or healthy control samples tested positive, whereas four of the MS sera tested weakly positive (Fig. 1A). However, when subsequently examined by immunofluorescence for binding to HEK 293T cells transfected either with a codon-optimized XMRV Env expression construct or with the XMRV full-length VP62 molecular clone, these samples were found to be negative. As positive control, the polyclonal mouse antiserum used in the ELISA gave a strong signal in cells transfected with either expression vector (data not shown).

**Table 1:** Overview of patient samples and test results^1^.

Patient cohort	ELISA Env	ELISA Gag	IFA^2^	Nested PCR of PBMC DNA	RT activity in co-culture	Nested PCR of LNCaP DNA from co-culture
CFS	0/36	1/36	0/1	0/39	0/10	0/10
MS	4/112	0/112	0/4	0/50	n.d.	n.d.
Healthy	0/27	1/27	0/1	0/30	n.d.	n.d.

1 Number of positives/total number of patient samples tested.

2 Only samples above the ELISA cut-offs were assayed.

n.d.  =  not done.

Antibody responses to retroviral Gag proteins are frequently used to screen for infection. We therefore developed an antigen capture ELISA to detect XMRV Gag-specific antibodies that had a markedly reduced background compared to the previously used Gag-ELISA where antigen was adhered directly to the microtiter plate well [5]. The specificity and sensitivity of the new capture ELISA was demonstrated by a titration of a positive control goat serum (Fig. 1B). Using this assay, one of the 36 CFS patients examined, one of the healthy donors (Fig. 1C, upper panel) but none of the 112 MS patients (Fig. 1C, lower panel) were slightly reactive. However, these weakly positive sera failed to react with HEK 293T cells expressing the XMRV Gag protein or transfected with the XMRV VP62 molecular clone by immunofluorescence (data not shown). In summary, sera from neither 36 CSF and 112 MS patients nor from 27 healthy controls tested positive for antibodies to XMRV Env and Gag proteins (Table 1).

### Provirus PCR in cultured PBMC

**Figure 2:**
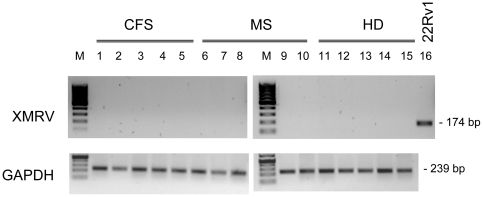
Diagnostic PCR of activated PBMC from CFS and MS patients and healthy donors. Representative results of nested XMRV PCRs with template DNAs from activated PBMCs after 7 days of culture. Samples from 5 CFS patients (lanes 1–5), 5 MS patients (lanes 6–10) and 5 healthy donors (HD, lanes 11–15) are shown. 200 ng of human genomic DNA spiked with 14 pg of 22Rv1 DNA (genome equivalent of approximately two cells) were used as positive control (lane 16). Results of corresponding single round PCRs for GAPDH as control for DNA integrity and absence of PCR inhibitors are shown in the lower panel. M  = 100 bp marker.

MLVs and other closely related retroviruses are unable to cross the nuclear pore complex for integration into the genomic DNA of the host, relying instead on mitosis to access the host cell chromosomes [18]. In order to improve the sensitivity of the test we therefore activated PBMC with PHA and cultured in the presence of IL-2 for seven days prior to DNA isolation, a process likely to increase the number of XMRV-infected cells. PBMC from 39 CFS patients, 50 multiple sclerosis patients with high fatigue scores (FSS median 4.7, range 2.9-7.0) and 30 healthy volunteers were analyzed, of which the corresponding sera from 36 CFS patients, all 50 MS patients and 17 of the 30 healthy donors had already been tested by ELISA (Fig. 1). The integrity of the isolated DNA and the absence of PCR inhibitors were confirmed by amplification of GAPDH sequences (Fig. 2). PBMC culture and DNA preparations were performed in laboratories of the Charité clinic and the PCRs were run at the Robert Koch Institute in a blinded manner. First, nested PCRs to detect proviral XMRV *gag* sequences with high sensitivity and specificity [5] were run in parallel by two operators in different laboratories in a blinded fashion and all DNA samples were found to be consistently negative (Table 1, see Fig. 2 for representative results). Furthermore, when DNA samples from a random selection of 13 CFS and 20 healthy donor were tested using an alternative nested PCR described by Urisman *et al* [1] the results were also negative (data not shown).

### Co-culture of PBMC with LNCaP cells

**Figure 3:**
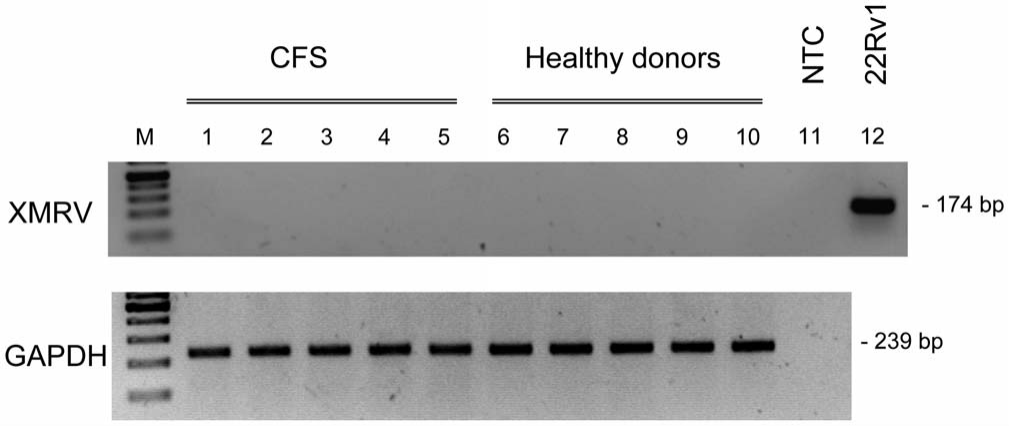
Lack of infection of XMRV susceptible LNCaP cells by co-culture with activated PBMCs. PCR results with isolated LNCaP cell DNA after co-culture with PBMCs from CFS patients (lane 1–5) and healthy donors (lanes 6–10). Five representative samples out of 10 co-cultures for each group are shown. As control, LNCaP cells were infected with XMRV-containing supernatant from 22Rv1 cells (lane 12). A water-only control (no template control, NTC) was run in lane 11. Results of the GAPDH PCR with the same samples are shown in the lower panel. M  = 100 bp marker.

Patient cells were cocultivated with the highly susceptible prostate cancer cell line LNCaP [19] in an attempt to expand, as reported by Lombardi *et al* [7], possible low levels of infection. Activated PBMC from 10 randomly chosen CFS patients and from 10 healthy donors were added at day three to LNCaP cells for three hours to allow virus transmission. Supernatant from chronically infected 22Rv1 cells was added to separate LNCaP cell cultures as a positive control. After seven days, DNA was isolated from the LNCaP cells and tested by nested XMRV-Gag PCR [5]. The PBMC from neither the 10 CFS patients nor the 10 healthy donors transmitted XMRV to the LNCaP cells (Table 1), whereas incubation with 22Rv1 supernatant resulted in the expected infection of the indicator cells (Fig. 3). Contamination of this DNA preparation with murine genomic DNA or with the pcDNA3.1-VP62 plasmid was ruled out using a PCR designed to detect murine DNA (kindly provided by B. Klempa) and a nested-PCR able to detect less than 10 copies of the pcDNA3.1-VP62 plasmid in 200ng of human DNA (data not shown). This control was necessary because the plasmid is used frequently in many laboratories including our own. Consistent with the PCR results, RT activity was only detected in the supernatants of LNCaP cells incubated with 22Rv1 cell supernatant (Table 1).

### Infection of PBMCs with XMRV

We next addressed the question as to whether PBMCs from CFS patients and healthy donors are susceptible to XMRV infection and if so, whether infectious virus is released. At day 3 post-activation, PBMCs from a randomly selected subset of 5 CFS patients and 5 healthy donors were incubated with cell-free supernatant from 22Rv1 cells or with the same supernatant previously subjected to heat inactivation. All supernatants were treated with DNase I to exclude carryover of 22Rv1 cell genomic DNA. LNCaP cells were also incubated with the supernatants as a positive control.

**Figure 4:**
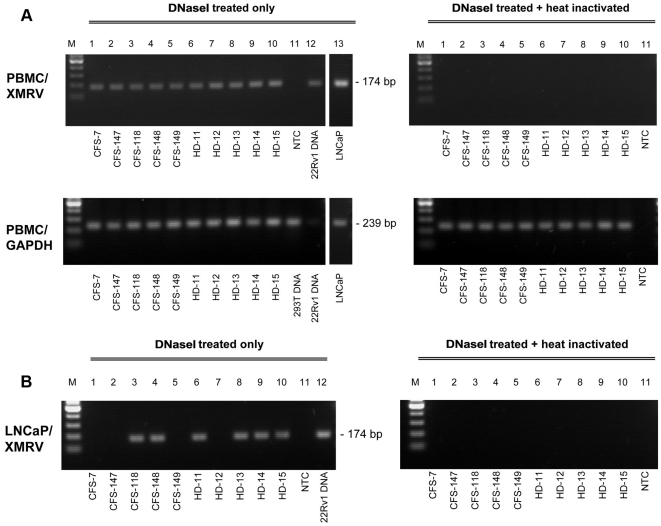
Human PBMC cultures are susceptible to productive infection by XMRV. (A) Detection of proviral XMRV sequences by nested PCR in DNAs of PBMC from 5 CSF patients (lanes 1–5) and 5 healthy donors (HD, lanes 6–10) infected for a week with DNase I treated (left hand panel) or DNase I treated and heat-inactivated (right hand panel) supernatants from the XMRV producing 22Rv1 cell line. The no template control (NTC) is in lane 11. DNA prepared from 22Rv1 cells was used as a positive control (lane 12, left panel). Results of a single round PCR for GAPDH are depicted underneath. (B) On the day of DNA isolation, supernatants from the infected PBMC cultures were used to test for virus transmission to LNCaP indicator cells. The results of a nested XMRV PCR with DNA prepared from the exposed LNCaP cells a week after incubation with the PBMC supernatants are shown (lanes 1–10). The control set up was the same as described above (lanes 11 and 12). M  = 100 bp marker. The entire experiment has been repeated twice with similar results.

After seven days in culture, DNA was isolated and subjected to diagnostic nested PCR. XMRV provirus was detected in DNA from each of the 10 infected PBMC samples, whereas the samples incubated with heat-inactivated virus remained negative (Table 2 and Fig. 4A). DNA integrity was confirmed by amplification of the GAPDH gene and no contamination with mouse genomic DNA or with the pcDNA3.1-VP62 plasmid was detected. RT activity, indicating the release of viral particles, was detected in 9/10 supernatants (4/5 CFS and 5/5 healthy donors) collected at 7 days post infection from the infected PBMC (Table 2). Except one CFS patient (CFS-7) which supernatant was RT negative the measured RT activities in PBMC supernatants of infected CFS patients and healthy donors were between 136 and 1218.5 µU/ml (median of 469 µU/ml). Taking into account the 1∶100 pre-dilution, 945.3 mU/ml of RT activity were present in the supernatant of the infected LNCaP cells. Thus, PMBC either from CFS patients or from healthy donors released about 2000-fold less viral particles than LNCaP cells in our setting. Supernatants of PBMCs or LNCaP cells incubated with heat-inactivated 22Rv1 supernatant remained negative for RT activity. Therefore, PBMCs can be infected by XMRV and cells carrying the provirus are present and release viral particles after a week.

**Table 2:** Infection of human blood cells with XMRV from the 22Rv1 cell line.

		PBMC culture		LNCaP culture	
	Infection with:	Diagnostic PCR	RT-activity	Diagnostic PCR	RT-activity
CFS patients	SN	5/5	4/5	2/5	2/5
	SN heat inact.	0/5	0/5	0/5	0/5
Healthy donors	SN	5/5	5/5	4/5	3/5
	SN heat inact.	0/5	0/5	0/5	0/5

SN =  Supernatant.

Finally, susceptible LNCaP cells were exposed to PBMC supernatants taken at 7 days post infection to determine whether the newly produced virus particles are themselves infectious. XMRV could be detected by diagnostic PCR in DNA isolated from LNCaP cells a week after exposure to supernatants from XMRV-infected PBMC from 2/5 CFS patients and 4/5 healthy donors (Table 2 and Fig. 4B). Supernatants from none of the PBMC cultures that were initially incubated with heat inactivated 22Rv1 supernatants were able to infect LNCaP cells. The integrity of the isolated LNCaP cell DNA was also confirmed by GAPDH amplification and the absence of XMRV plasmid DNA and mouse genomic DNA was demonstrated by PCR (data not shown). Furthermore, RT activity was detected in the supernatant from 5 of the 6 LNCaP cell cultures that tested positive for XMRV infection by PCR (Table 2). In summary, these results demonstrate that cells present in human PBMC cultures can be productively infected by XMRV and release infectious virus particles.

## Discussion

CFS/ME is a multifaceted illness with an unknown etiology and a diagnosis based on the careful exclusion of other conditions and diseases with similar symptoms including HIV infection and multiple sclerosis [9], [20]. Recently, the novel human retrovirus XMRV was reported to be detectable in the lymphocytes of two-thirds of American patients fulfilling the 1994 international CFS case definitions [8] and the 2003 Canadian Consensus Criteria, as well as in lymphocytes from almost 4% of healthy controls [7]. This association raises the obvious possibility that XMRV may be a causative agent of CFS or a contributing factor in the pathogenesis of the disease. Furthermore, the authors found evidence for an XMRV-specific antibody response in infected CFS patients, a finding that would be expected as no closely related endogenous human retroviral sequences are known that might induce tolerance. Specific antibodies to the virus have also been identified in the sera from XMRV-positive prostate cancer patients [2] and more recently in experimentally infected macaques [21].

Interestingly, patients suffering from the common neuroimmunologic disorder multiple sclerosis [10] also show fatigue symptoms that closely resemble in part those of CFS. Although this MS-related fatigue [11] is a major contributor to the reduced quality of life suffered by MS patients, the biological cause remains unknown. However, the similar clinical presentation of CFS and MS-related fatigue suggests a possible common etiology. That retroviruses may be an underlying cause for the neuroimmunological dysfunctions that appear with both diseases has already been proposed [22], [23], [24], [25]. In this regard it should also be kept in mind that HTLV I is able to induce Tropical Spastic Paraparesis (TSP) a neuronal degenerative disease in some of the infected individuals and leukemia or lymphomas in others [26]. We therefore attempted to find evidence for XMRV infection of CFS and MS patients by screening their sera by ELISA. The CFS patients, recruited from the Charité, were diagnosed according to the 1994 international CFS case definitions. And the MS patients, recruited from the Charité's NCRC outpatient clinic, were diagnosed with definite relapsing remitting multiple sclerosis according to the current panel criteria [27]. In addition to the 36 CFS patients and 112 MS patients, 27 healthy individuals donated serum for testing. Using a previously described and evaluated Env-ELISA [5], [17] and a newly developed capture Gag-ELISA we found no evidence for specific seroreactivity among any of the human samples screened. These results are in stark contrast with the original description of a significant association between XMRV-infection and CFS [7] but are in agreement with a more recent study in which plasma samples from CFS cases in the USA were tested and found to be negative [17].

In addition to the serological assays, we used nested PCR to test DNA from *in vitro* activated PBMCs for the presence of proviral XMRV. Activation by PHA and culture in the presence of IL-2 is likely to increase the number of infected cells by stimulating virus expression and the infectivity of the cells, thereby increasing the detection sensitivity. Indeed, expression of XMRV protein has been described in activated T- and B-cells [7]. Nevertheless, even after stimulation for seven days, none of the 119 PBMC cultures yielded detectable XMRV sequences (39 from CFS patients, 50 from MS patients and 30 from healthy blood donors). Four previous studies have already reported negative results using DNA isolated from the unstimulated PBMCs of more than 410 CFS patients [13], [14], [15], [17], contradicting the hypothesis that most individuals affected by CFS carry the virus. Moreover, in a recent report addressing XMRV in Danish patients with MS no XMRV has been found as well. In this study single-round PCRs for *gag* and *env* sequences with DNA from unstimulated PBMC were used [28]. Our nested PCR set up consistently detects 10 proviral XMRV sequences in 200-800ng of human DNA [5] and as 200ng DNA corresponds to a genome equivalent of about 33,300 cells (6pg DNA/cell), the test, performed in duplicate, has a detection limit of one provirus in at least 3,300 PBMC. It should be noted that although a very low ratio of infected cells could result in inconsistent or even negative PCR results, such low ratios would be inconsistent with published data demonstrating that a considerable proportion of activated PBMCs from XMRV-positive CFS patients express viral proteins, detected by intracellular flow cytometry [7]. To minimize the possibility of failing to detect a provirus due to primer mismatch, 33 samples were also run with the nested PCR primers and conditions previously published by Urisman *et al* [1]. However, this PCR setup also failed to yield positive results. Furthermore, co-cultures of activated PBMCs with XMRV permissive LNCaP cells yielded neither free virus in the supernatants (RT assay) nor proviral DNA in the LNCaP cells (nested PCR), despite the transmission of XMRV from PBMCs to LNCaP cells having been documented [7].

A very recent publication reports finding MLV-like *gag* sequences in 32 out of 37 (86.5%) CFS patients and in 3 out of 44 (6.8%) healthy blood donors [29]. These sequences, amplified from the DNA of unstimulated, uncultured PBMC using single round or nested PCR with previously described XMRV primer sets [1], [7] were clearly distinct from XMRV, being more closely related to modified-polytropic and polytropic murine endogenous retroviruses than to XMRV itself. Although the selection of primers for the nested PCR used in our study were primarily guided by the sequences of known XMRV isolates, an alignment with murine endogenous retroviruses genomes similar to the one used by Urisman *et al*. [1] has been done to match with most conserved regions in the *pre*-*gag*/*gag* region. Our intention was to take advantage of the XMRV distinctive 24 nt deletion in this region in order to discriminate between XMRV and other MLVs on the size of the PCR product. According to sequence homology our PCR set up should therefore amplify many of the known polytropic, modified-polytropic and xenotropic proviruses in addition to XMRV. Nevertheless, we can presently not formally exclude that a proportion of the non-XMRV proviruses that have been amplified in the report by Lo and co-workers [29] could have been missed using our primer set.

The failure to detect XMRV in donor PBMCs prompted us to test PBMCs from a subset of the CFS patients and healthy donors for susceptibility to infection with the virus. Incubation with XMRV from the persistently infected 22Rv1 cell line resulted in the infection of all PBMC cultures. To avoid false-positive results we also tested samples giving a positive XMRV signal for contamination with murine genomic DNA or with plasmids containing the VP62 molecular clone. Indeed, this was done for all intentionally infected PBMC and LNCaP cultures and PBMC/LNCaP co-cultures.

Although we did not ourselves investigate the phenotype of the blood cells being infected with XMRV, Lombardi *et al.* have reported the infection of T- and B-lymphocytes [7]. Infected blood cells release only very low levels of virus, barely detectable with the highly sensitive RT assay we used, whereas in contrast the supernatants of infected LNCaP cells generally contained levels of RT activity higher by 3 - 4 orders of magnitude. Nevertheless, one week after infection, the supernatants from over half of the PMBC cultures contained sufficient virus to infect LNCaP cells. The relatively poor growth in PBMCs compared with that in the type I IFN-insensitive LNCaP cells could due to a low LTR promoter activity [30], [31] and/or from the action of cellular antiretroviral responses. It was recently reported that XMRV does not possess countermeasures against the IFN-induced membrane protein tetherin [32], and that therefore the release of virions from human PBMC is probably inhibited by the action of this human protein. Furthermore, this and other papers demonstrated that the human protein APOBEC 3G, also expressed in human PBMC but not in LNCaP cells, inhibits XMRV replication [32], [33], [34]. An abundant particle production and rapidly spreading XMRV infection in human PBMC cultures therefore seems unlikely and is not supported by our data. Peripheral blood mononuclear cells are preferred targets for many retroviruses including MLV and additional experiments are needed to determine the precise replication kinetics of XMRV in human PBMC cultures and the subpopulation of blood cells involved.

Taken together we have used ELISA, sensitive PCR and co-culture with permissive indicator cells in an attempt to detect XMRV infection in cohorts of CFS and MS patients (with or without MS related fatigue) and healthy controls. No evidence of infection was found in any of the serum or activated PBMC samples. However, we could confirm that PBMC cultures from healthy donors and from CFS patients can be experimentally infected with XMRV and produce transmissible virus.

## Methods

### Study population and specimen collection

CFS patients were diagnosed at the outpatient Clinic for Adult Immunodeficiencies of the Dept. of Medical Immunology at the Charité between 2007 and 2009. Diagnosis was based on a thorough clinical evaluation of patients who had suffered from sudden onset of severe fatigue for at least 6 months, had no historical, laboratory or diagnostic evidence of exclusionary medical, neurological or psychiatric disease and fulfilled the Fukuda criteria (Fukuda 1994). A total of 39 CFS patients (13 males and 26 females, ranging from 17 to 56 years of age (median age 41)) were included in the study.

MS patients were recruited from the Charité's NCRC outpatient clinic. Neurological disability was assessed using Kurtzke's Expanded Disability Status Scale (EDSS) [35] (39 males and 73 female patients, ranging from 20 to 54 years of age (mean age 39); EDSS ranging from 0.0 to 6.0 (median EDSS 2.0)). All patients had a defined relapsing remitting MS according to the current panel criteria [27]. MS related fatigue, assessed using the Fatigue Severity Scale (FSS [36]), was diagnosed in 56 of the 112 patients (median FSS score = 4.0 (range 1.0–7.0), with a score above 4 defining fatigue).

Heparinized blood samples from patients and from age-matched healthy donors (taken during the same period) were processed to yield PB mononuclear cells (PBMC) by density gradient centrifugation using Ficoll-Hypaque and cryopreserved until analysis.

All patients gave written informed consent for the use of their samples to test etiological theories of CFS and MS, respectively, and the study was approved by the Ethics Committee of the Charite - Universitätsmedizin Berlin.

### Enzyme Linked Immunosorbent Assays

The solid phase ELISA for the detection of anti-XMRV Env antibodies has been described elsewhere [5]. Briefly, bacterially expressed and purified (via His-tag) XMRV envelope proteins were coated overnight on Probind-96-well plates (Becton Dickinson Labware Europe, Le Pont de Claix, France) at room temperature in equimolar amounts. The plates were blocked with 100 µl 2% Marvel milk powder in phosphate buffered saline (PBS) for 2h at 37°C, washed three times with PBS containing 0.05% Tween 20 (Serva, Heidelberg, Germany) and 50 µl mouse sera or patient sera at a 1∶200 dilution in PBS containing 2% milk powder and 0.05% Tween 20 (PMT) were added to each well. After incubation for 1 hour at 37°C, plates were washed three times and 50 µl of a 1∶1000 dilution of a goat anti-mouse IgG-HRP conjugate or goat anti-human IgG-HRP conjugate (both Sigma Aldrich, Munich, Germany) in PMT was added. After further incubation for 1 hour at 37°C, plates were washed three times and 50 µl substrate solution (0.6mg/ml ortho-phenylendiamin (OPD) in 0.05 M phosphate-citrate buffer, pH 5.0 containing 0.4 µl/ml of a 30% hydrogen peroxide solution) was added. After 5–10 minutes the color development was stopped by the addition of 20 µl 1M sulfuric acid and the absorbance at 492nm was measured using a microplate reader.

For detection of antibodies against XMRV Gag a capture ELISA was developed. 96-well plates were coated overnight with 0.3 µg per well of purified monoclonal antibody R187 (kindly provided by N. Fischer, Hamburg, Germany). A solution containing 25ng/well of the C-terminal half of the recombinant XMRV Gag protein [5] was added. Plates were incubated for 1h and after washing three times the sera samples were applied as described above. Goat polyclonal antisera (kindly provided by B. Switzer, CDC, Atlanta, USA) specific for MLV p30 and Friend MLV [17], detected with rabbit anti-goat HRP conjugate (DAKO, Hamburg, Germany) at a 1∶5000 dilution, were used to validate the assay.

### Cell culture and antibody purification

The human prostate cancer cell line LNCaP (ATCC #CRL-1740), the XMRV positive human prostate cancer cell line 22Rv1 [37] (ATCC #CRL-2505) and the MLV p30 specific antibody producing hybridoma cell line R187 (ATCC #CRL-1912) were grown in RPMI 1640 (Gibco) supplemented with 10% FCS (Biochrome TPP). The HEK 293T cell line was grown in DMEM (Gibco) supplemented with 10% FCS. Stored PBMC were thawed and analyzed for viability by trypan exclusion staining. PBMC were cultured in 6-well plates (1×10^6^ cells per ml) for seven days at 37°C in a humidified 5% CO_2_ atmosphere using Iscoves medium (Biochrome Berlin, Germany) containing 10% AB serum or 20% FCS, rhIL-2 (180 IU/ml, R&D Systems) and PHA (4 µg/ml).

For co-cultivation, 1ml suspensions of 1×10^6^ PBMC activated for 3 days were added to 40–60% confluent LNCaP cells in 6-well plates and removed 3h later by washing. LNCaP cells were cultured for a further seven days in media without IL-2 and PHA to avoid persistence of residual PBMC. Supernatants for measurement of RT activity were taken on day 7.

Human PBMC were infected with XMRV-containing supernatant from 22Rv1 cells. Briefly, fresh supernatant from 22Rv1 culture was centrifuged, sterile filtered and treated with RNase-free DNase I (Qiagen, Hilden, Germany) according to the manufacturer's instructions but omitting the final DNase heat-inactivating step. This DNase treatment was included to avoid possible carry-over of XMRV provirus-containing genomic DNA from the 22Rv1 cells. One aliquot of the supernatant was heat inactivated at 60°C for 1h and used as a control. 3×10^6^ PBMC were pelleted and resuspended in 200 µl of the supernatants and after 2h incubation at 37°C the cells were washed twice with PBS and cultivated in 12-well plates for seven days.

Antibodies were purified from supernatant of R187 hybridoma cells using a protein G Sepharose 4 Fast Flow column (GE Healthcare Europe, Munich, Germany) according to the manufacturer's instructions.

### PCR

Tissue culture, DNA preparation and PCR testing were carried out in different laboratories by different operators. Briefly, DNA was extracted from PBMCs after seven days of cultivation with IL-2 and PHA using the Qiagen Blood DNA Minikit (Qiagen, Hilden, Germany), aliquoted and stored at −20°C. Nucleic acid concentrations were determined using a Nanodrop device (Fisher Scientific, Schwerte, Germany).

Diagnostic nested PCRs were performed as described elsewhere [1], [5] using 200ng –800ng template DNA per reaction and the primer pairs *Out-For* (5′-CCGTGTTCCCAATAAAGCCT-3′) and *Out-Rev* (5′-TGACATCCACAGACTGGTTG-3′) or *In-For* (5′-GCAGCCCTGGGAGACGTC-3′) and *In-Rev* (5′-CGGCGCGGTTTCGGCG-3′), respectively The integrity of the DNA samples was checked using a GAPDH PCR as described previously [38]. The PCRs were run at least twice and by two different operators in physically isolated rooms to avoid contamination. In order to rule out the presence of potentially contaminating VP62 plasmid DNA, a highly sensitive nested PCR using forward primers within the pcDNA3.1 backbone vector and reverse primers within XMRV was carried out. Primers used for the outer PCR were *CMV-For* (5′-AACCCACTGCTTACTGGCTTATCG-3′) and *In-Rev* (5′- CGGCGCGGTTTCGGCG -3′), for the inner PCR *T7-For* (5′- AATACGACTCACTATAGGG -3′) and *Deletion-Rev* (5′- CCCCAACAAAGCCACTCCAAAA -3′). Conditions for the first round PCR were 95°C/10min, 95°C/30sec, 60°C/30sec, 72°C/1min for 20 cycles and a final elongation of 72°C/5min. Using 1/10^th^ (2.5 µl) of the first reaction the second round PCR was performed as describe above but with an annealing temperature of 55°C and for 35 cycles.

To screen for potential contamination with murine genomic DNA a PCR designed to amplify a fragment of the rodent mitochondrial DNA (D-loop) was also carried out. Primer sequences CB1n (5′-GGAGGMCARCCAGTWGAAYACCCATT-3′) and 12S1n (5′-TAATTATAAGGCCAGGACCAAACCT-3′) were kindly provided by B. Klempa (Charité, Berlin, Germany) and PCR conditions were 95°C/10min, 95°C/30sec, 66°C/30sec, 72°C/1.5min for 35 cycles and a final elongation of 72°C/5min.

### Immunofluorescence microscopy

Cells were grown on gelatine (0.3% coldwater fish gelatine in distilled water) coated glass slides in 12-well plates and 24h after seeding were transfected using Polyfect Reagent (Qiagen) with the full length molecular clone pcDNA3-VP62 or with the pTH-XMRV-coEnv or pTH-XMRV-coGAG plasmids containing codon optimized synthetic full-length genes of the XMRV *env* or *gag* under control of the CMV promoter. 48h after transfection the cells were fixed with 2% formaldehyde (Sigma) in PBS and the glass slides were placed upside-down on microscopy slides. Cells were rinsed briefly in PBS, permeabilized with 0.5% Triton X-100 in PBS for 15 min and washed 3 times with PBS. After 30 min incubation with blocking buffer (2% Marvel milk powder in PBS) cells were incubated for 60 min at 37°C with the positive control mouse Gag- or Env-antisera or human sera diluted 1∶200 in blocking buffer. The slides were washed extensively with PBS and the secondary antibodies conjugated to fluorophores were added for 30 min. After thorough washing steps with PBS, the cells were mounted in Mowiol. Images were obtained on a Zeiss (LSM510) confocal laser-scanning microscope.

### RT activity assay

Quantification of type C retroviral reverse transcriptase activity was performed using the Mn^2+^ C-type activity Kit (Cavidi, Uppsala, Sweden) according to the manufacturer's instructions. Specific RT activity and background were determined using the Mn^2+^ reverse transcriptase supplied with the kit. Cell culture supernatants were sampled on the days stated and remaining cells or debris removed by centrifugation. Samples were stored at −80°C before being thawed and tested in (at least) duplicate.
